# Protocol for the differentiation of induced pluripotent stem cells into retinal pigment epithelial cells

**DOI:** 10.1016/j.xpro.2025.104263

**Published:** 2025-12-08

**Authors:** Mark Zorin, Rike Ewerling-Hähnel, Kerstin Nagel-Wolfrum

**Affiliations:** 1 Institute of Developmental Biology and Neurobiology, Johannes Gutenberg University of Mainz, 55128 Mainz, Germany; 2 Institute of Molecular Physiology, Johannes Gutenberg University of Mainz, 55128 Mainz, Germany

**Keywords:** Cell culture, Developmental biology, Cell differentiation

## Abstract

Induced pluripotent stem cells (iPSCs) are emerging as a valuable system for modeling tissues and organs. Here, we describe a highly scalable protocol for the differentiation of iPSCs into retinal pigment epithelium (RPE), including a new step that makes it easier for researchers to obtain a high-purity culture. We also describe a cryopreservation technique for RPE progenitor cells to enable their storage. The use of cryopreserved cells allows a subsequent reduction in differentiation time compared to the full protocol.

## Highlights

• Steps for the differentiation of iPSCs into retinal pigment epithelial cells
• Steps for cryopreservation of RPE progenitors
• Reduction in differentiation time using cryopreserved RPE progenitors

## Abstract (editor highlights)

Publisher’s note: Undertaking any experimental protocol requires adherence to local institutional guidelines for laboratory safety and ethics.

## Graphical abstract

**Figure:**
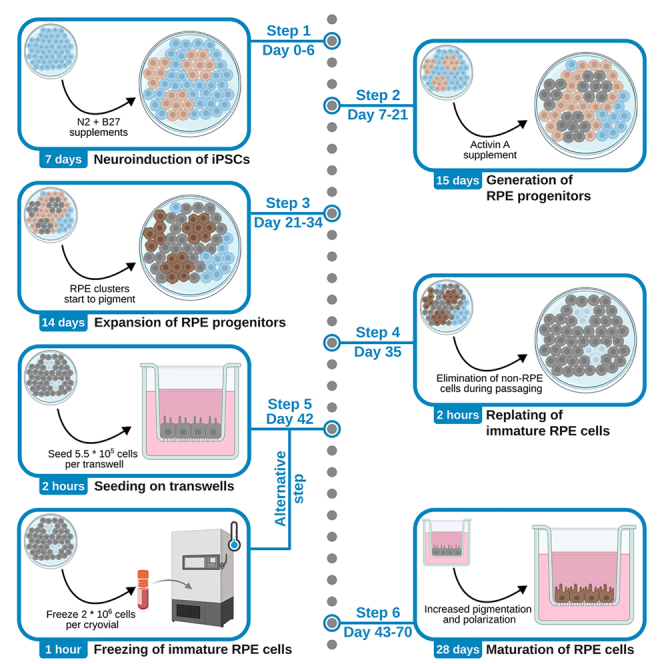

## Before you begin

Before you start, you should obtain an iPSC culture by purchasing an established cell line from a cell bank or by reprogramming cells, such as human dermal skin fibroblasts or lymphoblasts by yourself. We perform episomal reprogramming of fibroblasts into human iPSCs by transfecting the cell using the Yamanaka factors OCT3/4, SOX2, KLF4, L-MYC and the embryonic stem cell specific microRNA miR302/367.^1^ iPSCs should be carefully characterized, including testing for the most common copy number variations, performing embryoid body assay formation followed by immunocytochemistry and in case of patient-derived cells, Sanger sequencing to determine the type of disease-causing mutations.

### Innovation

Here we describe an improved protocol of the differentiation of human induced pluripotent stem cells (iPSCs) from healthy donors into retinal pigment epithelium (RPE), based on previously published protocols.^1^^,^^2^^,^^3^ This article introduces new adaptation steps for cell medium composition changes and streamlines the differentiation process by integrating additional steps that enable high-purity RPE culture to be obtained. Additionally, we provide a cryopreservation technique that allows researchers long-term storage of RPE progenitors. These cells can be utilized in subsequent differentiation rounds, thereby significantly reducing the overall differentiation time. Moreover, we have also used this protocol to generate RPE cells from patient-derived iPSCs with different disease-causing pathogenic variants, and it has proven to be robust and efficient.

### Institutional permissions (if applicable)

The use of human fibroblasts, the generation of iPSC and the differentiation into RPE may need to be approved by your local ethics committee.

## Key resources table

**Table:** 

REAGENT or RESOURCE	SOURCE	IDENTIFIER
**Antibodies**		

Rabbit polyclonal anti-ZO-1 (Zonula Occludens-1), working dilution 1 in 1000	Proteintech	Cat#21773-1-AP
Mouse monoclonal anti-Ezrin, working dilution 1 in 200	Sigma-Aldrich	Cat#E8897
Mouse monoclonal anti-PMEL (premelanosome protein), working dilution 1 in 50	Invitrogen	Cat#MA5-13232
Mouse monoclonal anti-ARL13B (ADP-ribosylation factor-like protein 13B), working dilution 1 in 200	Abcam	Cat#ab136648
Alexa Fluor 647-conjugated donkey anti-mouse IgG, working dilution 1 in 400	Invitrogen	Cat#A-31571
Alexa Fluor 568-conjugated goat anti-mouse IgG, working dilution 1 in 400	Invitrogen	Cat#A-11031
CF 640R-conjugated donkey anti-rabbit IgG, working dilution 1 in 200	Biotrend	Cat#20178

**Chemicals, peptides, and recombinant proteins**		

mTeSR Plus Basal Medium (mTeSR)	STEMCELL Technologies	Cat#100-0274
DMEM/F12 + GlutaMAX	Gibco	Cat#31331028
Neurobasal medium	Gibco	Cat#21103049
DMEM high glucose + GlutaMAX	Gibco	Cat#31966021
KO (Knockout) serum replacement	Gibco	Cat#10828010
Geltrex (working dilution 1:100 in DMEM)	Gibco	Cat#A1413202
DPBS (no calcium, no magnesium)	Gibco	Cat#14190094
Gentle Dissociation Buffer	Gibco	Cat#1315014
Accutase solution	Sigma-Aldrich	Cat#A6964
ROCK inhibitor	STEMCELL Technologies	Cat#72302
B27 supplement	Gibco	Cat#17504044
N2 supplement	Gibco	Cat#17502048
2-Mercaptoethanol	Gibco	Cat#31350010
Penicillin-Streptomycin	Gibco	Cat#15140122
MEM NEAA	Gibco	Cat#11140050
Bovine Serum Albumin (BSA)	Millipore	Cat#126609-5GM
Recombinant human Activin A	Proteintech	Cat#HZ-1138
TRIzol Reagent	Invitrogen	Cat#15596026
GoScript Probe 2-step RT-qPCR system	Promega	Cat#A5000
iTaq Universal SYBR Green Supermix	Bio-Rad	Cat#1725124

**Experimental models: Cell lines**		

Induced pluripotent stem cells generated from healthy donors	Schwarz et al.^1^	N/A

**Software and algorithms**		

Fiji software	National Institutes of Health	Website
GraphPad Prism 8	GraphPad Software	Website

**Oligonucleotides**		

Primer: GAPDH Forward: GAGTCAAGGGATTTGGTCGT	N/A	N/A
Primer: GAPDH Reverse: TTGATTTTGGAGGGATCTCG	N/A	N/A
Primer: RPE65 Forward: GTCTATCCAGGTTGAGCATCC	N/A	N/A
Primer: RPE65 Reverse: CCACATCGAAGGAGACTGC	N/A	N/A
Primer: BEST1 Forward: TCATCCCCATTTCCTTCGTG	N/A	N/A
Primer: BEST1 Reverse: TTGCTCGTCCTTGCCTTC	N/A	N/A
Primer: RLPB1 Forward: GAGAGGGTGCAAGAGAAGG	N/A	N/A
Primer: RLBP1 Reverse: CTGTCAAAGAGCTCAGGGTAC	N/A	N/A

**Other**		

Transparent TC-inserts for 12-well plate (transwells; pore size: 0.4 μm)	Sarstedt AG & Co. KG	Cat#83.3931.041
Cell strainer (pore size: 100 μm)	Falcon	Cat#10737821
Mowiol	Carl Roth GmbH & Co. KG	Cat#0713.1
cellZscope2	nanoAnalytics	N/A
NanoDrop 2000c Spectrophotometer	Thermo Fisher Scientific	Cat#ND-2000C
QuantStudio RealTime PCR system	Applied Biosystems	N/A
Photoreceptor outer segments (POS) labeled with FITC	Plaza Reyes et al.^4^	N/A

## Materials and equipment

**Table:** Neuroinduction basal medium

Reagent	Final concentration	Amount
DMEM/F12 + GlutaMAX	50%	237.2 mL
Neurobasal medium	50%	237.2 mL
2-Mercaptoethanol	55 μM	550 μL
GlutaMAX	1x	5 mL
Penicillin-Streptomycin	1x (1%)	5 mL
**Total**	**N/A**	**485 mL**

**CRITICAL:** After preparing Neuroinduction basal medium, store 48.5 mL aliquots at −20°C.

***Note:*** Use the aliquot of Neuroinduction basal medium within 2 weeks after thawing.

**Table:** Neuroinduction complete medium

Reagent	Final concentration	Amount
Neuroinduction basal medium	1X	48.5 mL
B27 supplement	1x	1 mL
N2 supplement	1x	500 μL
**Total**	**N/A**	**50 mL**

**CRITICAL:** Prepare 1 mL aliquots of B27 supplement, 500 μL aliquots of N2 supplement separately and freeze them at −20°C.

**CRITICAL:** After addition of B27 and N2 supplements, keep Neuroinduction complete medium at +4°C for a maximum of 2 weeks. A color change during storage, indicative of a pH level shift, should prompt the preparation of fresh medium.

**Table:** RPE medium

Reagent	Final concentration	Amount
DMEM high glucose + GlutaMAX	1x	440 mL
KO serum replacement	10%	50 mL
MEM NEAA	1x	5 mL
2-Mercaptoethanol	55 μM	550 μL
Penicillin-Streptomycin	1x (1%)	5 mL
**Total**	**N/A**	**500.55 mL**

**CRITICAL:** After preparing RPE medium, store 45 mL aliquots at −20°C.

***Note:*** Use the aliquot of RPE medium within 1 month after thawing.

**CRITICAL:** Reconstitute 10 μg of recombinant human Activin A in 100 μL sterile DPBS containing 0.1% Bovine serum albumin. Prepare aliquots (2 μL) with concentration 0.1 mg/mL and freeze them at −20°C. Avoid following freeze and thaw cycles.

**CRITICAL:** Avoid using Activin A aliquots older than 6 months.

**Table:** Freezing medium

Reagent	Final concentration	Amount
KO serum replacement	90%	900 μL
DMSO	10%	100 μL
**Total**	**N/A**	**1 mL per cryovial**

***Note:*** Do not store Freezing medium. Prepare it according to the number of vials that you will require.

## Step-by-step method details

### Seeding iPSCs for differentiation (day −2)

**Timing: 2 h**

Before starting to differentiate iPSCs into RPE, the iPSCs should be seeded into the wells of a 6-well plate.

**Figure 1:**
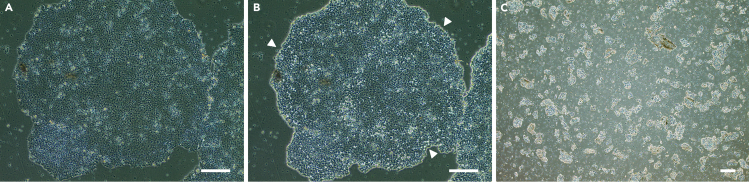
Seeding of iPSCs for differentiation (A) Colony of iPSCs before dissociation under phase contrast. (B) Colony of iPSCs after 2 minutes of incubation with Gentle Dissociation Buffer. The arrowheads point at the colony’s marginal cells which after dissociation have a distinctive shape and become brighter under phase contrast. (C) Dissociated colonies of iPSCs before seeding on the coated well for differentiation. Cell clumps after resuspending should be roughly the same size. Scale bars: 200 μm.
  c. Add 1 mL of Gentle Dissociation Buffer and incubate the cells 1–3 minutes at room temperature.
  d. Aspirate the Gentle Dissociation Buffer and add 1 mL of mTeSR.
  e. Check under the microscope whether the iPSCs colonies appear brighter under phase contrast light and whether the marginal cells have a distinctive shape (Figure 1B).
  f. Dissociate the cells using a cell scraper and resuspend them 3–5 times to obtain the cell clumps of similar small size (Figure 1C).
  g. Based on individual characteristics of the cell line, seed the appropriate number of the cells on the coated wells (see Critical note below). Culture the cells in 1.5 mL of mTeSR with ROCK inhibitor at final concentration of 10 μM.
  h. Place cells in a 37°C-5% CO_2_ incubator.

1. Prepare the new culturing plate:
  a. Coat the wells of a 6-well plate with 1 mL of Geltrex and incubate for at least 1 hour at 37°C-5% CO_2_ incubator.
  b. After 1 hour of incubation, remove the Geltrex and wash the wells with 1 mL of sterile DPBS.
  c. After washing, add 1 mL of fresh mTeSR with 10 μM ROCK inhibitor and preheat the plate in a 37°C-5% CO_2_ incubator before seeding the cells.
2. Seeding the cells:
  a. Remove mTeSR medium from culture plate by gentle aspiration.
  b. Wash cells with 2 mL sterile DPBS (Figure 1A).

**CRITICAL:** Ensure that all cells are seeded at a concentration that will allow the cells to form a 70%–90% confluent monolayer within two days.

**CRITICAL:** At Step 2f, a single-cell suspension can be made. The choice of the seeding method may depend on the particular characteristics of the cell line involved.

### Maintaining of the iPSC culture (day −1)

**Timing: 30 min**

This section describes iPSCs maintaining steps.

**Figure 2:**
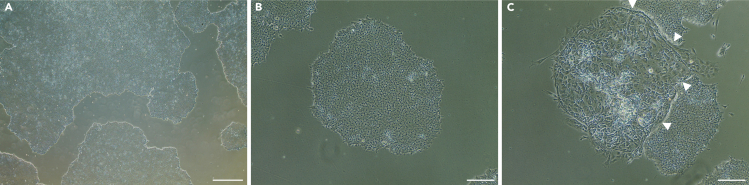
Maintaining of the iPSCs culture (A) Representative phase contrast image showing an 80% confluence layer of the iPSCs which can be used to initiate RPE differentiation. Scale bar: 500 μm. (B and C) Representative phase contrast images of (B) a standalone iPSCs colony with no sings of spontaneous differentiation and (C) a spot with prominent uncontrolled differentiation. Arrowheads are pointing towards the boarder between iPSCs colonies and differentiated cells. Scale bars: 200 μm.

***Note:*** If the cells reached the 80%–90% confluence on Day −1, move on directly to Step 4 (Figure 2).

3. Change media:
  a. Remove the medium by gentle aspiration.
  b. Add 1.5 mL mTeSR medium for further cultivation.
  c. Place cells in a 37°C-5% CO_2_ incubator.

### Production of neural clusters (day 0–5)

**Timing: 6 days**

In this step, the neuronal clusters are formed. They will serve as a source of RPE progenitors.

**CRITICAL:** Cells layer should be 80%–90% confluent (Figure 2A) and show no signs of spontaneous differentiation (Figures 2B and 2C).

4. Prepare Neuroinduction complete medium:
  a. Thaw and warm up the aliquot of Neuroinduction basal medium at room temperature.
  b. Add 1 mL of B27 and 500 μL of N2 supplements, mix well.
5. Start Neuroinduction:
  a. Remove mTeSR by gentle aspiration.
  b. Wash cells twice with 2 mL sterile DPBS.
  c. Gently add 4 mL Neuroinduction complete medium.
  d. Place cells in a 37°C-5% CO_2_ incubator.

**CRITICAL:** On Day 2 and Day 4, remove medium from the cells by gentle aspiration and cautiously add 4 mL fresh Neuroinduction complete medium. During the medium change **avoid** washing steps with DPBS, as it could lead to cell detachment.

**CRITICAL:** Pre-warm Neuroinduction basal or complete medium to room temperature before use. Take care during media change as the cells can easily detach.

***Note:*** Use the aliquot of Neuroinduction basal medium within 2 weeks after thawing.

***Note:*** Until Day 5 there could be a massive cell death, and the medium may rapidly turn yellow.

### Gradual medium change (day 6)

**Timing: 1 h**

On Day 6, the Neuroinduction complete medium is partially replaced by RPE medium. As the composition of these media is completely different, this step serves to adapt the cells to the new conditions.

6. Make 50% Neuroinduction complete medium + 50% RPE medium mix:
  a. Thaw and warm up the aliquot of RPE medium at room temperature.
  b. Mix 1 mL of Neuroinduction complete medium + 1 mL RPE medium. Total amount of medium mix is 2 mL per well.
7. Perform a medium change:
  a. Gently aspirate the Neuroinduction complete medium from the 6-well plate.
  b. Add the mixture of Neuroinduction complete medium + RPE medium prepared on Step 6.
  c. Place cells in a 37°C-5% CO_2_ incubator.

**CRITICAL:** Be very careful when changing the media as the cells can easily detach.

***Note:*** On Day 6, there could be a massive cell death, and the medium may rapidly turn yellow.

### Generation of RPE progenitor clusters (day 7–21)

**Timing: 15 days**

**Figure 3:**
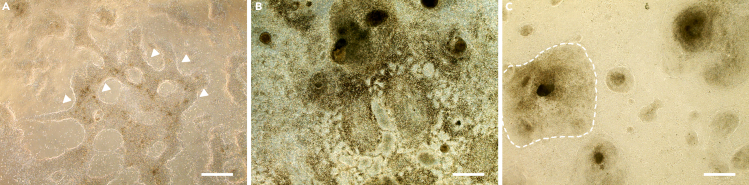
Generation of RPE progenitor clusters (A) Representative phase contrast image of RPE progenitors clusters on Day 8 of the differentiation. The arrowheads point at the borders of these clusters. (B and C) On Day 21, the pigmented spots can be observed by eye. They could form (B) a monolayer or (C) be localized in clusters. The RPE progenitor clusters are surrounded with dotted lines. Scale bars: 500 μm.

***Note:*** Instead of forming a monolayer, cells could form clusters of pigmented cells (Figure 3C). This is a normal differentiation process for some cell lines.

From Day 7 to Day 21 the clusters of RPE progenitors will be formed (Figure 3A). To promote the cells forming the RPE progenitor clusters, add Activin A to the RPE medium.

8. Medium change on Day 7:
  a. Combine in a 15 mL centrifuge tube RPE medium (4 mL per well) and Activin A to the final concentration 50 ng/mL and mix well.
  b. Gently aspirate the culturing medium in the 6-well plate.
  c. Carefully add 4 mL RPE medium with Activin A per well.
9. Change the RPE medium with Activin A 3 times a week (on Monday, Wednesday and Friday) and incubate cells in a 37°C-5% CO_2_ incubator.

***Note:*** Until Day 9, there could be a massive cell death, and the medium may rapidly turn yellow.

**CRITICAL:** The first signs of the pigmentation that can be visible by eye should appear around Days 18–21 (Figures 3B and 3C). When pigmentation appears, culture cells in RPE medium without Activin A. Proceed directly to Step 10.

**CRITICAL:** If there is no pigmentation visible on Day 22 the differentiation has failed, and the cells can be discarded.

### Expansion of RPE progenitor clusters (day 21–34)

**Timing: 14 days**

From Day 21 to Day 34 the RPE progenitors start to mature, and the pigmented area becomes larger. During this step, culture cells in RPE medium.

10. Change the RPE medium 3 times a week (on Monday, Wednesday and Friday), apply 4 mL RPE medium per well of a 6-well plate.

***Note:*** On Day 35, the pigmented area should cover approximately 40%–50% of the well surface.

### Passaging of RPE progenitors (day 35)

**Timing: 2 h**

**Figure 4:**
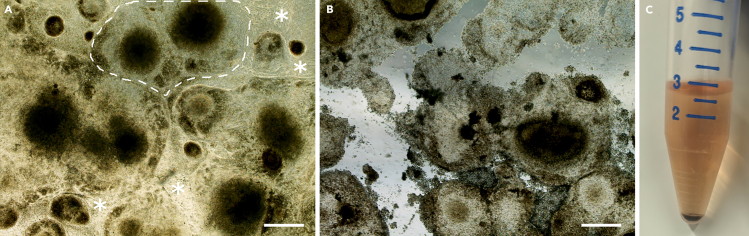
Passaging of the RPE progenitors Representative images of the colonies of the RPE progenitors under phase contrast (A) before and (B) after applying Gentle Dissociation Buffer. RPE clusters are surrounded with dotted lines, while the non-RPE monolayer is highlighted by asterisks. (C) The black pellet contains the pigmented RPE progenitors, obtained on Step 13k. Scale bars: 500 μm.

On Day 35, the cell layer contains clusters of RPE progenitors and non-RPE cells (Figure 4A). To enhance differentiation efficiency and culture purity, non-RPE cells should be removed, allowing for the enrichment of RPE progenitors.

11. Warm RPE medium, DPBS, Gentle Dissociation Buffer and Accutase to room temperature.
12. Prepare a new 6-well plate:
  a. Coat the wells with 1 mL of Geltrex for 1 hour at 37°C-5% CO_2_ incubator. For one well of RPE progenitors approximately 3 new wells are needed.
  b. After 1 hour, aspirate Geltrex and wash the wells with 1 mL of DPBS.
  c. Add 2 mL of fresh RPE medium and leave the wells in the incubator until needed (Step 13m).
13. Passaging of RPE progenitors:
  a. Aspirate the RPE medium and wash the cells with 2 mL of sterile DPBS.
  b. Aspirate DPBS, add 1 mL of Gentle Dissociation Buffer per well and incubate the plate at 37°C-5% CO_2_ for 5 minutes.
  c. Take the plate out from the incubator and start tapping gently to detach non-RPE cells (Figure 4B).
  d. Aspirate Gentle Dissociation Buffer containing detached non-RPE cells.
  e. Apply 1 mL of Accutase per well and place the plate at 37°C-5% CO_2_ for
  10–15 minutes.
  f. After incubation, detach RPE progenitor cells with a cell scraper or by pipetting.
  g. Resuspend the cells several times to break up clumps to obtain a uniform single-cell suspension.
  h. Wash a cell strainer with DPBS to wet the entire surface of the strainer.
  i. Filter the cell suspension through the cell strainer and collect cells into 15 mL centrifuge tube.
  j. Add 2 mL of RPE medium in the well to collect the remaining cells and filter the cell suspension through the cell strainer that was used on the previous step.
  k. Centrifuge the cells at 0.4 RCF for 5 minutes (Figure 4C).
  l. Aspirate the supernatant, resuspend cells in 3 mL fresh RPE medium and count them.
  m. Seed 2∗10^6^ cells per well on Geltrex-coated 6-well plate. The culturing volume of RPE medium is 4 mL per well.
  n. Place cells in a 37°C-5% CO_2_ incubator.

***Note:*** The adhesion of RPE progenitors is much stronger compared to non-RPE cells. Therefore, only non-RPE cells are detached in Steps 13b-d and the RPE progenitors are dissociated in Steps 13e-f.

**CRITICAL:** If many cells from RPE progenitor clusters detach at Step 13c, proceed directly to Step 13f.

***Note:*** At Step 13k, a dark cell pellet should form after centrifugation. The intensity of the color of the pellet is a good indicator of the quality of the differentiation. Darker pellets generally indicate better differentiation (Figure 4C).

### Cultivation of immature RPE cells (day 36–41)

**Timing: 6 days**

During this step, RPE progenitors begin to divide and undergo the first steps of maturation.

14. Change the RPE medium 3 times a week (on Monday, Wednesday and Friday), the culturing volume per well is 4 mL.

**Figure 5:**
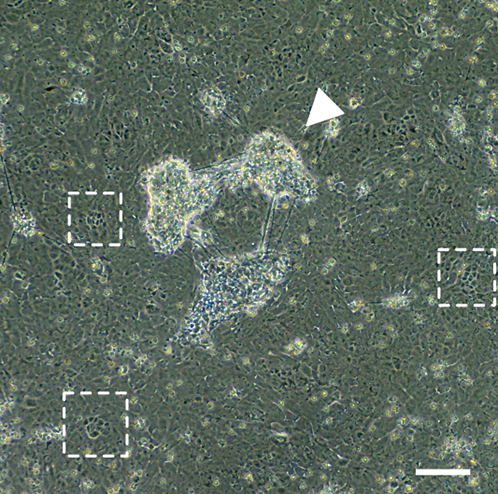
Cultivation of immature RPE cells Representative phase contrast image of the monolayer of immature RPE cells on Day 41 with 3D structure formed by non-RPE cells (arrowhead). The RPE cells undergo the first steps of maturation, become smaller and display a hexagonal shape (these clusters are marked by dotted lines). Scale bar: 300 μm.

***Note:*** Until Day 41, the remaining non-RPE cells will form small 3D spheres on top of the monolayer of immature RPE cells (Figure 5). These spheres can be easily removed on Day 42.

### Seeding of RPE cells on transwells (day 42)

**Timing: 2 h**

On Day 42, the cell layer contains immature RPE cells with a small contamination of other cell types, which should be removed from the culture to obtain better differentiation results.

***Note:*** Depending on culture purity, more or fewer non-RPE clusters may be observed.

15. Warm RPE medium, DPBS, Gentle Dissociation Buffer and Accutase up to room temperature.
16. Prepare P-12 transwells:
  a. Coat the transwells with 200 μL of Geltrex for 1 hour at 37°C-5% CO_2_ incubator. One well of 6-well plate of immature RPE can be seeded onto approximately 6 transwells (inserts for a 12 well plate).
  b. After 1 hour, aspirate Geltrex and add 500 μL of DPBS per well. Leave the wells in the 37°C-5% CO_2_ incubator until they are needed (Step 17n).
17. Passaging of immature RPE cells:
  a. Aspirate the RPE medium and wash the cells with 2 mL of sterile DPBS per well.
  b. Aspirate DPBS and add 1 mL of Gentle Dissociation Buffer per well and incubate the plate at 37°C-5% CO_2_ for 5 minutes.
  c. Take the plate out from the incubator and start tapping gently to get rid of spheres of non-RPE cells.
  d. Aspirate Gentle Dissociation Buffer containing spheres of non-RPE cells.
  e. Apply 1 mL of Accutase per well and place the plate at 37°C-5% CO_2_ incubator for 10–15 minutes.
  f. After incubation, detach the cells using a cell scraper or by pipetting.
  g. Resuspend the cells several times to break up clumps to obtain a uniform single-cell suspension.
  h. Wash the cell strainer with DPBS to wet the entire surface of the strainer.
  i. Filter the cell suspension through the cell strainer and collect cells into 15 mL centrifuge tube.
  j. Add 2 mL of RPE medium in the well to collect the remaining cells, resuspend the cell suspension and filter it through the cell strainer.
  k. Centrifuge the cells at 0.4 RCF for 5 minutes.
  l. Discard the supernatant, resuspend cells in fresh RPE medium and count the cells.
  m. Carefully aspirate DPBS from the transwells.
  n. Seed 5.5∗10^5^ cells per transwell. Fill the upper chamber with RPE medium up to 300 μL. The lower chamber volume is 900 μL.
  o. Place cells in a 37°C-5% C0_2_ incubator.

**CRITICAL:** If many cells detach at Step 17c, proceed directly to Step 17f.

***Note:*** The number of cells required for seeding may vary depending on the size of the transwell used. The general rule is to seed 5∗10^5^ of immature RPE cells per 1 cm^2^ of a transwell.^2^

***Note:*** At Step 17k, a dark cell pellet should form, but the color of the pellet would be much brighter compared to Step 13k.

### Alternative step 1: Freezing immature RPE cells (day 42)

**Timing: 1 h**

On Day 42, rather than seeding the cells on transwells, it is possible to freeze the immature RPE cells in order to utilize them in subsequent differentiation cycles starting on Day 42 (Alternative Step 2, Step 23).

18. Warm RPE medium, DPBS, Gentle Dissociation Buffer, Accutase, KO serum replacement and DMSO up to room temperature.
19. Prepare the desired number of cryovials (approximately 2 vials per well of 6-well plate) and a freezing box with 2-Propanol.
20. Prepare the Freezing medium that contains 90% KO serum replacement and 10% of DMSO.
21. Passaging of immature RPE cells for further cryopreservation:
  a. Aspirate the RPE medium and wash the cells with 2 mL of sterile DPBS per well.
  b. Aspirate DPBS and add 1 mL of Gentle Dissociation Buffer per well and incubate the plate at 37°C-5% CO_2_ incubator for 5 minutes.
  c. Take the plate out from the incubator and start tapping gently to get rid of spheres of non-RPE cells.
  d. Aspirate Gentle Dissociation Buffer containing spheres of non-RPE cells.
  e. Apply 1 mL of Accutase A per well and place the plate at 37°C-5% CO_2_ incubator for 10–15 minutes.
  f. After incubation, detach the cells using a cell scraper or by pipetting.
  g. Resuspend the cells several times to break up clumps to obtain a uniform single-cell suspension.
  h. Wash the cell strainer with DPBS to wet the entire surface of the strainer.
  i. Filter the cell suspension through the cell strainer and collect cells into 15 mL centrifuge tube.
  j. Add 2 mL of RPE medium in the well to collect the remaining cells, resuspend the cell suspension and filter it through cell strainer.
  k. Centrifuge the cells at 0.4 RCF for 5 minutes.
  l. Discard the supernatant, resuspend cells in fresh RPE medium and count the cells.
  m. Centrifuge the cells at 0.4 RCF for 5 minutes.
  n. Discard the supernatant, re-suspend the cells in the Freezing medium to a final concentration 2∗10^6^ cells per mL.
  o. Distribute the cell suspension into cryovials, 1 mL per vial.
  p. Freeze the vials in the freezing container overnight at −80°C. On the next day transfer the cells to the liquid nitrogen for long-term storage.

**CRITICAL:** If many cells detach as a sheet or as separate cells at Step 21c, proceed directly to Step 21f.

### Alternative step 2: Thawing the immature RPE cells (day 42)

**Timing: 1 h**

After thawing the cells from the liquid nitrogen, they should be cultured in a well of a 6-well plate for 1 week before being seeded onto transwells. The overall time of the differentiation is extended by 7 days.

22. Warm up RPE medium and DPBS to the room temperature and warm up a water bath to 37°C.
23. Prepare the 6-well plate for culturing:
  a. Coat the wells of a 6-well plate with 1 mL of Geltrex for 1 hour at 37°C-5% CO_2_ incubator. Seed one vial of frozen immature RPE cells on 1 well of a 6-well plate.
  b. After 1 hour, aspirate Geltrex and add 1 mL of DPBS to each well. Leave the plate in the incubator until required (Step 24e).
24. Thaw the immature RPE cells:
  a. Take the vial from a liquid nitrogen and place it in the pre-warmed water bath for
  2–3 minutes until it is fully thawed.
  b. Transfer the cell from the cryovial into 15 mL centrifuge tube. Wash the remaining cells in the vial with 1 mL of RPE medium and add it into centrifuge tube.
  c. Centrifuge the tube at 0.4 RCF for 5 minutes.
  d. Gently aspirate the supernatant and resuspend the pellet in RPE medium.
  e. Seed the cell from one vial on 1 well of 6-well plate (2∗10^6^ cells per well). The RPE medium volume is 4 mL per well.
  f. Place cells in a 37°C-5% C0_2_ incubator.

### Alternative step 3: Cultivation of immature RPE cells (day 43–48)

**Timing: 6 days**

During this step, the immature RPE cells will start to divide and show the first signs of maturation.

**Figure 6:**
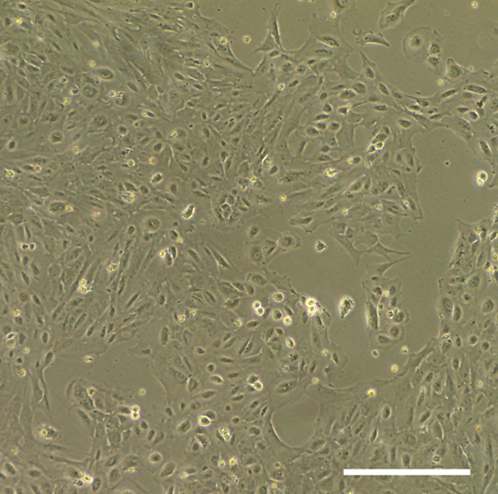
Thawing the immature RPE cells Representative phase contrast image of immature RPE cells taken the next day after thawing (Step 24). The cells exhibit a distinctive fibroblast-like shape. Scale bar: 300 μm.

***Note:*** On Day 43 the immature RPE cells will have a fibroblast-like morphology (Figure 6). After forming a monolayer, they start to decrease in size, and some cell clusters will show hexagonal morphology (Figure 5).

25. Change the RPE medium 3 times a week (on Monday, Wednesday and Friday), the volume per well is 4 mL. Cells are incubated in a 37°C-5% CO_2_ incubator

***Note:*** Until Day 48, the non-RPE cells will form small 3D spheres on top of the monolayer of immature RPE cells (Figure 5). These spheres can be easily removed on Day 49 (Alternative step 4).

### Alternative step 4: Seeding of RPE cells on the transwells (day 49)

**Timing: 2 h**

The cell layer on Day 49 contains immature RPE cells and a small contamination of other cell types, which should be removed from the culture to obtain better differentiation results.

26. Warm RPE medium, DPBS, Gentle Dissociation Buffer and Accutase up to room temperature.
27. Prepare P-12 transwells:
  a. Coat the transwells with 200 μL of Geltrex for 1 hour at 37°C-5% CO_2_ incubator. One well of immature RPE can be split on approximately 6 transwells (the inserts for a 12 well plate).
  b. After 1 hour aspirate Geltrex and add 500 μL of DPBS per well. Leave the wells in the incubator until they are needed (Step 28n).
28. Passaging of immature RPE cells:
  a. Aspirate the RPE medium and wash the cells with 2 mL of sterile DPBS per well.
  b. Aspirate DPBS and add 1 mL of Gentle Dissociation Buffer per well and incubate the plate at 37°C-5% CO_2_ incubator for 5 minutes.
  c. Take the plate out from an incubator and start tapping gently to get rid of spheres of non-RPE cells.
  d. Aspirate Gentle Dissociation Buffer containing spheres of non-RPE cells.
  e. Apply 1 mL of Accutase per well and place the plate at 37°C-5% CO_2_ incubator for
  10–15 minutes.
  f. After incubation, detach the cells using a cell scraper or by pipetting.
  g. Resuspend the cells several times to break up clumps to obtain a uniform single-cell suspension.
  h. Wash the cell strainer with DPBS to wet the entire surface of the strainer.
  i. Filter the cell suspension through the cell strainer and collect cells into 15 mL centrifuge tube.
  j. Add 2 mL of RPE medium in the well to collect the remaining cells, resuspend the cell suspension and filter it through cell strainer.
  k. Centrifuge the cells at 0.4 RCF for 5 minutes.
  l. Discard the supernatant, resuspend cells in fresh RPE medium and count the cells.
  m. Carefully aspirate DPBS from the transwells.
  n. Seed 5.5∗10^5^ cells per transwell. Fill the upper chamber with RPE medium to 300 μL. The lower chamber volume is 900 μL.
  o. Place cells in a 37°C-5% C0_2_ incubator.

**CRITICAL:** If the cells detach as a sheet at Step 28c, proceed directly to Step 28f.

***Note:*** The number of cells required for seeding may vary depending on the size of the transwell used. The general rule is to seed 5∗10^5^ of immature RPE cells per 1 cm^2^ of a transwell.^1^

***Note:*** Instead of seeding RPE cells on the transwells, you could isolate their RNA, DNA or proteins to use this expression level as a baseline for your follow-up investigations.

### Maturation of RPE cells (day 43–70 or day 50–77 with alternative steps)

**Timing: 28 days**

During this step immature RPE cells will undergo the maturation process.

**Figure 7:**
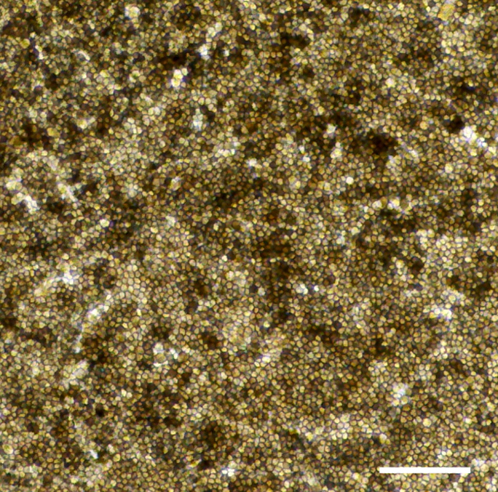
Morphology of mature RPE cells Representative phase contrast image of mature RPE cells showing a typical morphology of fully mature RPE cells, such as small cell size, hexagonal shape and high pigmentation rate. Scale bar: 300 μm.

29. Change RPE medium 3 times a week (on Monday, Wednesday and Friday). After 4 weeks on transwells the cells show the typical morphology of fully mature RPE cells: small cell size, hexagonal shape and high pigmentation rate (Figure 7).

**CRITICAL:** Record transepithelial electrical resistance (TEER) values at the end of each week of the maturation.

**CRITICAL:** The monolayer of RPE cells should be intact during maturation process. Be cautious during medium change to avoid scratching the cells sheet. The TEER measurements cannot be performed after monolayer scratching.

### Evaluation of RPE maturation process

The following section briefly describes possible methods to evaluate and quantify the success of differentiation of iPSCs into mature RPE cells. In addition to the approaches mentioned, further experiments can be performed, such as flow cytometry, scanning electron microscopy, or ELISA to assess the polarization of the cells, secretion of vascular endothelial growth factor-A (VEGF-A) and pigment epithelium-derived factor (PEDF).^5^^,^^6^

### Transepithelial electrical resistance measurement

**Figure 8:**
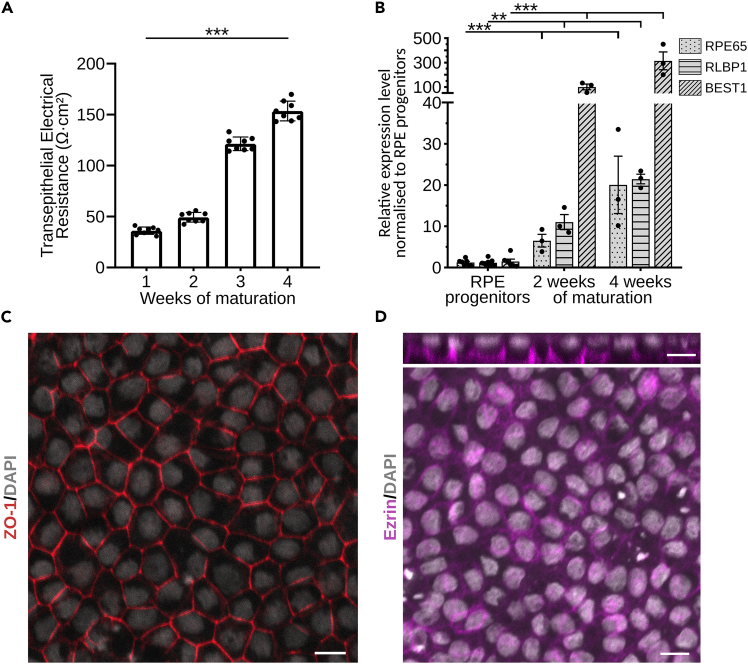
Characterization of RPE (A) Bar plot represents the values of transepithelial electrical resistance of RPE monolayer during 4 weeks of maturation. The data is represented as mean of 3 independent biological replicates with technical replicates depicted as dots ± SD. Statistical analysis was done using unpaired Student’s *t* test ∗p ≤ 0.05, ∗∗p ≤ 0.01, ∗∗∗p ≤ 0.001. (B) Bar plot represents a change in expression levels of *RPE65*, *RLBP1* and *BEST1* genes, canonical markers of mature RPE cells. The samples after 2 and 4 weeks of maturation are compared to the progenitor state of the cells. The data is represented using mean value of 3 independent biological replicates ± SEM. Outliers were excluded using the ROUT method (Q = 5%). Statistical analysis for mRNA analysis is based on delta CT values. Statistical analysis was done using one-way analysis of variance (ANOVA) followed by Dunnett’s multiple comparison test; ∗p ≤ 0.05, ∗∗p ≤ 0.01, ∗∗∗p ≤ 0.001. (C) Immunofluorescence image of mature RPE cells stained for a tight junction marker ZO-1 (red) and nuclei (DAPI, grey). Scale bar: 10 μm. (D) Immunofluorescence image of mature RPE cells stained for a marker of apical microvilli Ezrin (magenta) and nuclei (DAPI, grey). The top image represents an orthogonal projection of the same frame, showing localization of Ezrin staining on the apical side of cells. Scale bars: 10 μm.

The transepithelial electrical resistance (TEER) is a non-invasive method to quantitatively assess the integrity and barrier function of cell monolayers. TEER values reflect the presence and tightness of tight junctions between neighboring cells and serve as a functional indicator of epithelial maturity.^7^ In the context of RPE maturation, higher TEER values correlate with increased tight junction formation and thus a more mature, polarized monolayer.^8^^,^^9^^,^^10^ Monitoring TEER across differentiation experiments ensures consistency, reproducibility and comparable maturation levels. In this protocol, TEER measurements were performed using the cellZscope2 device (Figure 8A).

### Quantitative RT-PCR analysis of canonical RPE markers

To monitor the maturation dynamics of RPE cells during the cultivation on transwells, quantitative PCR (qPCR) can be performed to assess the expression of canonical RPE markers such as *RPE65*, *RLBP1* and *BEST1*. RNA is typically isolated at three key time points during maturation: from RPE progenitors before seeding them on transwells, after 2 and 4 weeks of culture on transwells. A significant increase in expression levels of canonical RPE markers compared to progenitor cells is expected over time, indicating progressive maturation (Figure 8B).

### Characterization of morphological features via immunocytochemistry

**Figure 9:**
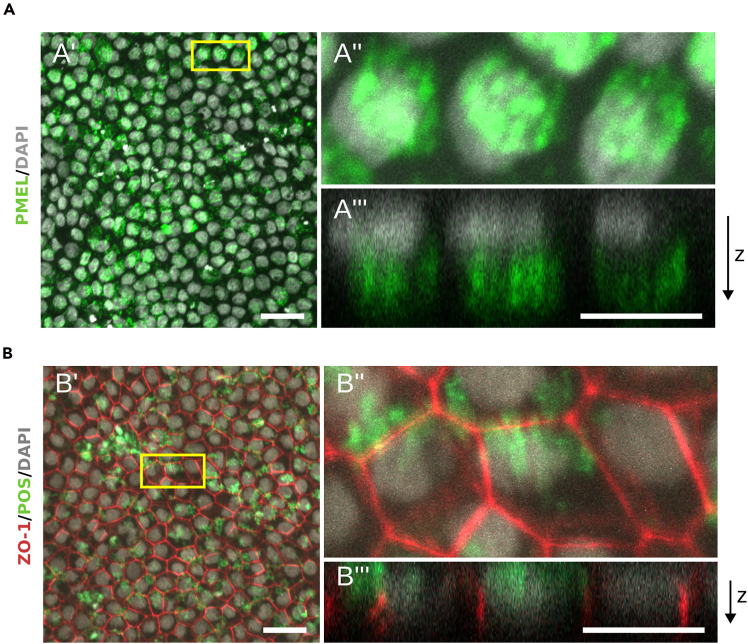
Functional characterization of mature RPE cells (A) Immunofluorescence image of mature RPE cells (A′) stained for PMEL (green) with DAPI (grey) co-staining. Scale bar: 25 μm. The selected region (A″) was used to create an orthogonal projection of a z-stack (A‴), that shows PMEL signals localized at the apical side of the cells, opposite to the basal side where nucleus is located. Scale bar: 10 μm. (B) Immunofluorescence image of mature RPE cells (B′) stained for ZO-1 (red) and nuclei (DAPI, grey) after exposition to bovine photoreceptors outer segments coupled with Alexa 488 (POS, green) for 6 hours. (B″) The selected region from B’. Indirect immunofluorescence double staining for ZO-1 (red), photoreceptors outer segments (green) and nuclei of RPE cells with DAPI (grey). Scale bar: 25 μm. (B‴) The selected region (B″) was used to create an orthogonal projection of a z-stack, that shows the fluorescent signal from internalized POS in relation to the nuclei. Scale bar: 10 μm.

Immunocytochemistry of RPE markers allows visualization of protein expression and localization within cells, confirming that key RPE-specific proteins are produced and properly distributed. This technique verifies the structural and functional maturation of RPE cells, reflecting their characteristic morphology. In this protocol, the tight junction protein Zonula Occludens-1 (ZO-1), the apical microvilli marker Ezrin (Figures 8C and 8D) and the premelanosome protein (PMEL) (Figure 9A) were used as markers of mature RPE cells.

### Functional characterization of mature RPE cells via phagocytosis assay

One of the main features of the RPE cell *in vivo* is the daily phagocytosis of photoreceptor outer segment tips (POS), an essential process for maintaining retinal homeostasis.^11^ To assess the functionality and maturation stage of the RPE cells *in vitro*, a phagocytosis assay can be performed. In this protocol, cells that were cultured for 4 weeks on transwells are used to assess POS uptake, confirming the acquisition of key physiological properties characteristic of native RPE (Figure 9B). This assay is also suitable for identifying pathological phenotypes in patient-derived cellular models.

## Expected outcomes

After 70 days (or 77 days with alternative steps) the researchers should expect to have a fully mature RPE culture. The cells will reflect the gene expression profile, morphology and functionality of mature RPE cells from a human eye.^12^^,^^13^ Molecular markers for RPE can be analyzed by qPCR and immunofluorescence (Figures 8B–8D). Morphological characteristics could include: hexagonal shape (Figure 8C), high level of pigmentation (Figure 7), polarization of the cells, presence of microvilli on the apical part (Figure 8D). The functional characteristics are: the ability of phagocytosis, internalizing of photoreceptor outer segments (Figure 9B), and melanosomes trafficking to the apical part of the cell (Figure 9A).

## Limitations

This protocol is designed to be an easily scalable and cost-efficient way to differentiate iPSCs into a culture of high-purity RPE cells. Therefore, using a different type of stem cells, for example embryonic stem cells, may require an additional optimization of the differentiation protocol. In addition, there are several studies using the iPSC-derived RPE patches for transplantation into the eyes of animal models or humans, during clinical trials.^14^^,^^15^ In order to achieve the required level of purity of the RPE culture for transplantation purposes, it is necessary that researchers employ more sophisticated techniques.^3^ The present protocol was developed for the production of RPE from human iPSCs. In case non-human cells are used, the quality of differentiation might be affected or modifications in the protocol might be necessary. Researchers should note that iPSC-derived RPE cells could not fully reflect an *in vivo* state of the cells, due to the absence of a micro-environment, cell-cell contact between RPE cells and photoreceptors and gradients of small molecules and hormones. Current protocol has been successfully tested in four independent laboratories with seven different iPSC lines, from healthy donors, as well as from patients with various genetic backgrounds. However, researchers should be aware that poor-quality iPSCs, for example those with genomic rearrangements, will not differentiate successfully into RPE cells. Moreover, we assume that the genetic background of patients with certain diseases, e.g., ciliopathies, could have a negative effect on differentiation quality.

## Troubleshooting

### Problem 1

A low attachment rate or massive cell death of iPSCs is observed on Day −1. The confluency cannot be achieved in 2 days.

### Potential solution

• Some iPSC lines can be more sensitive to Gentle Dissociation Buffer, decrease the incubation time at Step 2c.
• Do not create a single cell suspension or seed a larger cell clumps at Step 2f.
• Consider seeding a larger number of iPSCs on Day −2.

### Problem 2

The cells die off or detach during the early steps of differentiation.

### Potential solution

• Carefully characterize the iPSCs before starting the differentiation protocol, as those with an altered karyotype usually fail to undergo differentiation.
• There should be no signs of differentiation on the monolayer of the iPSCs. In addition, check the cells for mycoplasma contamination beforehand.
• One potential explanation for the observed detachment could be attributed to an insufficient number of cells that are seeded in the beginning of Neuroinduction step. It is imperative to ensure that the differentiation process is initiated with an almost confluent layer of iPSCs.
• Be cautious when changing the medium before Day 9, during this time the cells can be easily washed away.

### Problem 3

On Day 21 there are no signs of pigmentation or there is a high level of contamination by non-RPE cells.

### Potential solution

• Make sure, that Activin A was stored in aliquots at −20°C, avoid freeze and thaw cycles.
• If there are no signs of pigmentation on Day 21, leave the cells in the culture for another 4–5 days. If the cells don’t start to pigment during this time, discard the cells and consider replacing an Activin A stock.
• If the selected iPSC clone still shows no pigmentation on day 21 after several rounds of differentiation, another iPSC clone of the same cell line should be used for differentiation.

### Problem 4

During passaging at Steps 13, 17, or 28, the RPE progenitor cells may detach, while non-RPE cells remain attached to the plate. In some cases, RPE progenitors can be detached using Gentle Dissociation Buffer during the passaging step.

### Potential solution

This issue can occur due to several factors, including iPSC clone characteristics, differentiation success, or disease-related phenotype. If the steps following Day 35 are successful, easily detachable RPE progenitors may not be a concern. However, if you encounter difficulties during subsequent differentiation steps, refer to the general recommendations in troubleshooting sections 1 and 2 for guidance on cell quality check.

### Problem 5

There are not enough cells for seeding on transwell or well of 6-well plate.

### Potential solution

• Check the general recommendations regarding quality of the cells at troubleshooting sections 1 and 2.
• Increase the number of wells that simultaneously undergo the differentiation and pull them together during Step 13 and 17 to achieve an expected cell density.

### Problem 6

The RPE monolayer is not getting darker during maturation stage (Day 43–70).

### Potential solution

• Check the general recommendations regarding quality of the cells at troubleshooting sections 1 and 2.
• Control the number of cells that were seeded on the transwell on Day 42 or Day 49. A low number of cells will slow down the maturation and pigmentation process.
• A low pigmentation could be considered as a disease specific phenotype.

## Resource availability

### Lead contact

Further information and requests for resources and reagents should be directed to and will be fulfilled by the lead contact, Kerstin Nagel-Wolfrum (nagelwol@uni-mainz.de).

### Technical contact

Technical questions on executing this protocol should be directed to and will be answered by the technical contact, Mark Zorin (mzorin@uni-mainz.de).

### Materials availability

Reagents and resources used in this study are commercially available. Nevertheless, requests for resources and reagents can be directed to the lead contact (nagelwol@uni-mainz.de) and copied to the technical contact (mzorin@uni-mainz.de).

### Data and code availability

This study did not generate new datasets.

## Acknowledgments

We thank H. May-Simera for kindly sharing the cellZscope2 and labeled photoreceptor outer segments. We thank Mike Cheetham for providing human iPSCs for protocol establishment. This work was supported by the German Research Council DFG SPP2127 “Gene and cell-based therapies to counteract neuroretinal degeneration” (NA 1398/1-2, number 399443882 [K.N.-W.]), MA 6139/3-1 399364546 (R.E.-H., HMS), seed funding as part of the Priority Programme SPP2127 (M.Z. and R.E.-H.), FAUN (K.N.-W.), Choroideremia Research Foundation (CRF), the Choroideremia Research Foundation Canada (CRFC), and Moon rocket Foundation (K.N.-W.).

## Author contributions

Conceptualization, M.Z. and K.N.-W.; investigation, M.Z. and R.E.-H.; writing – original draft, M.Z. and K.N.-W.; writing – review and editing, M.Z., R.E.-H., and K.N.-W.

## Declaration of interests

The authors declare no competing interests.
